# A meta‐analysis of self‐compassion and attachment in adults

**DOI:** 10.1111/papt.12590

**Published:** 2025-04-04

**Authors:** Charlotte Hill, Vasilis S. Vasiliou, Fuschia M. Sirois, Olivia Hughes, Andrew R. Thompson

**Affiliations:** ^1^ South Wales Clinical Psychology Training Programme, School of Psychology, Psychology Tower Building Cardiff University and Cardiff and Vale University Health Board Cardiff UK; ^2^ Department of Psychology, School of Life Sciences and the Environment Royal Holloway, University of London Egham Surrey UK; ^3^ Department of Psychology Durham University Durham UK

**Keywords:** attachment, attachment dimensions, meta‐analysis, moderators, self‐compassion

## Abstract

**Objective:**

Attachment might shape the extent to which a person is self‐compassionate. Despite the plethora of research examining attachment and self‐compassion, no previous systematic review has quantified the magnitude of the associations between self‐compassion and different attachment dimensions.

**Design:**

Random‐effects meta‐analyses examined the magnitude of the associations of self‐compassion with anxious, avoidant, and secure attachment, using correlational effects (*r*‐value). Moderator analyses tested whether the effects varied as a function of participant age, sex, population type (students vs. community sample) and attachment measure used within studies.

**Methods:**

A systematic search of the literature using SCOPUS, Web of Science, and CINAHL databases retrieved 37 eligible studies.

**Results:**

The meta‐analyses revealed a medium effect size for the positive association between self‐compassion with secure attachment, *r*_avg_ = .395, 95% CI [0.248, 0.524], and medium and small effect sizes for the negative associations with anxious attachment, *r*_avg_ = −.282, 95% CI [−0.329, −0.233], and avoidant attachment, *r*_avg_ = −.280, 95% CI [−0.320, −0.240]. Moderator analyses indicate that the magnitude of associations with avoidant attachment varied as a function of participant age and population type (students vs. community samples).

**Conclusions:**

The findings suggest differential associations between self‐compassion and attachment dimensions. Self‐compassion was positively associated with secure attachment, while the reverse was found for insecure attachment. Negative associations between self‐compassion and avoidant attachment were larger for older individuals. Ageing populations may be vulnerable to lower self‐compassion when already more prone to experiencing avoidant attachment. Compassion‐focused therapy may be an effective therapeutic option when working with individuals reliant on anxious or avoidant attachment dimensions.

## INTRODUCTION

Attachment plays an important role in an individual's development by shaping how they respond to interpersonal interactions and how they cope with life challenges and threats (Mikulincer & Shaver, 2007). People with secure attachment can foster self‐compassion as an effective mechanism that leads to successful coping with difficult events (Gilbert, 2014). Previous studies have provided a comprehensive synthesis of the association between self‐compassion and different dimensions of attachment (Lathren et al., 2021). A recent study has quantified the magnitude of associations between fears of self‐compassion with anxious and avoidant attachment, reporting moderate positive associations for both (Varley et al., 2024). To date, no quantitative synthesis of the association between self‐compassion and attachment exists to determine their average magnitude and indicate the factors that may attenuate or strengthen these associations. Equally, the extent to which individuals with different attachment dimensions, ages, or sexes utilise self‐compassion is less understood. These are important gaps to address, particularly considering that self‐compassion is a trainable skill (Kirby, 2017; Wilson et al., 2019), whilst conflicting theoretical perspectives are held about the stability of dimensions of attachment across time (Bowlby, 1988; Fraley, Vicary, et al., 2011). In the current meta‐analysis, we aim to address these gaps by systematically analysing findings from diverse studies to provide a clearer understanding of how dimensions of attachment influence the extent to which individuals can practice self‐compassion in times of distress, and explore the role of potential moderating factors, such as age and sex.

### The role of self‐compassion as a regulatory mechanism of attachment

Self‐compassion primarily acts as a regulatory response system (Inwood & Ferrari, 2018). Based on Neff's model (2003a), self‐compassion can be viewed as consisting of three bipolar dimensions: (i) treating oneself with kindness versus being self‐judgmental, (ii) seeing personal mistakes as common to humanity versus feeling isolated, and (iii) being mindful of one's feelings versus over‐identifying with them. Other models of self‐compassion, such as that described by Gilbert (2014) focus on how self‐compassion functions within a three‐part emotional regulatory system: soothe, threat, and drive responses (Depue & Morrone‐Strupinsky, 2005). The threat system can create a negative bias that helps people pay greater attention to negative events relative to positive ones, which likely serves an evolutionary function to establish safety and protection (Gilbert, 2014). The drive system strengthens individuals' behaviours to seek and acquire skills/resources to help them achieve goals, pleasure, and rewards. The soothe system fosters positive affect reflecting calmness, rest, and contentment. Gilbert (2014) proposes the need to activate self‐soothing to calm and balance the drive and threat responses to experience self‐compassion.

Multiple studies indicate that self‐compassion is a regulatory practice that is associated with psychological well‐being (Neff et al., 2007; Zessin et al., 2015), adaptive interpersonal relationships (Lathren et al., 2021), and improved physical health outcomes (Phillips & Hine, 2021). As self‐compassion influences self‐perception (Sirois, 2020) and regulates behaviours (Sirois et al., 2015), viewing it through an attachment style lens can help understand why individuals can use self‐compassion for their own well‐being (Zhang et al., 2022) while others struggle to do so (Allen et al., 1998). We propose that the study of attachment dimensions can shed more light on this.

### The intricate relationship of self‐compassion and dimensions of attachment

Bowlby (1969/1982) posits that individuals develop internal attachment styles through early bonding experiences and by gaining proximity with significant others (Bowlby, 1969; Wihelm et al., 2016). In adulthood, attachment styles reflect self‐perceptions and interpersonal relationships (Mikulincer, 1995) that can shape individuals' emotional, cognitive, and behavioural regulatory practices when interacting with close others (Shaver et al., 1996) or facing threats (Shaver & Mikulincer, 2002). There is consensus that adult attachment is likely shaped by early parent–child attachment (Fraley, 2002), although the degree of overlap between early attachment and adult romantic attachment, along with the degree of stability in dimensions of attachment across the lifespan, are more contentious topics across the literature (Fraley, 2018; Raque et al., 2023).

Self‐compassion is cultivated in positive family dynamics where caregivers are present, kind, and responsive (Moreira et al., 2018). For example, individuals growing up with a strong, supportive bond from caregivers, can present with a secure attachment style, and respond to threats with similar care towards themselves and others (Neff & McGehee, 2010). These responses reflect a healthy regulation of emotional development, showcasing resilience and heightened mindfulness (Neff & McGehee, 2010; Pepping et al., 2013, 2015). Additionally, self‐compassion seems to function as an adaptive emotion regulation strategy protecting individuals against the activation of problematic schemas or unhelpful beliefs about oneself and the world (Trompetter et al., 2017).

Individuals growing up with an inconsistent and unpredictable caregiving history can present with anxious attachment, and respond to threats with high anxiety and emotional volatility. These responses reflect a negative self‐view (Cantazaro & Wei, 2010; Pietromonaco & Barrett, 2000), self‐criticism (Bowlby, 1969/1982) and a pronounced need for external validation and reassurance (Wei et al., 2005). Therefore, people with anxious attachment dimensions may face challenges in emotional regulation (Mikulincer & Shaver, 2016) and exhibit a reduced inclination to extend kindness to themselves. Consequently, this hinders a person's ability to rely on their soothing emotional regulatory response to practice self‐compassion (Neff & McGehee, 2010). Due to persistent rejection and unresponsiveness from caregivers, people with avoidant attachment can develop a sense of self‐reliance (Mikulincer et al., 2003). While this self‐reliance may lead them to believe that they do not require support from others, it may also make individuals incapable of extending kindness and compassion to themselves (Joeng et al., 2017; Mackintosh et al., 2018).

### The role of contextual variables in influencing self‐compassion and attachment

Existing research suggests that contextual factors (such as demographics, e.g., age and sex) may have a potential moderating function in the link between self‐compassion and attachment. Studies indicate variability in attachment and self‐compassion separately in terms of sex differences, age differences, and differences in life experiences (Del Giudice, 2011; Pinquart et al., 2013; Yarnell et al., 2015). For example, men report higher self‐compassion than women in some studies (Muris et al., 2016; Yarnell et al., 2015). Research suggests that there is a higher prevalence of avoidant attachment in men, while women show a higher prevalence of anxious attachment (Del Giudice, 2011; Scharfe, 2016). Likewise, although weak associations between self‐compassion and age tend to occur in samples with limited variability, such as adolescents (Muris et al., 2016) or older adults (Phillips & Ferguson, 2013), moderate relationships are typically observed in multi‐generational samples (Wren et al., 2012). It may be that life events associated with different developmental stages influence attachment, suggesting that attachment may be a malleable construct to some extent (Fraley et al., 2020; Michael Bradley & Cafferty, 2001; Pinquart et al., 2013). Transitions to university/college have been considered important milestones showcasing some adaptation in attachment, where it presents a risk of shifting towards insecure dimensions (Lopez & Gormley, 2002). During this sensitive period, young adults often leave their family home and focus on developing their individuality, independence, and self‐identity (Hiester et al., 2009). However, this transition can also make them more susceptible to declines in mental health and psychological well‐being (Conley et al., 2014). Self‐compassion has been shown to help buffer against stressors associated with university/college on well‐being and to promote effective adjustment (Kroshus et al., 2021; Scott & Donovan, 2021). Examining the potential synergistic moderating function of contextual factors in the association between self‐compassion and attachment can provide clinically useful information for clinicians. Findings from such research can highlight how self‐compassion and attachment interact during stressful events, and how life experiences and an individual's stage in life influence these interactions. Research often relies on college and university populations, raising the question of whether this specific group serves as a contextual factor in the relationship between attachment and self‐compassion. Exploring this could provide valuable insights into the generalisability of findings to other populations.

### The problem of measures assessing attachment and self‐compassion

Multiple attachment measures are available that map onto different dimensional models of attachment (Fraley et al., 2000). A two‐dimensional lens (anxious and avoidant attachment) may be reflected within all measures of attachment, even across those originally based on a three‐dimensional understanding of attachment (Brennan et al., 1998b; Van Geel & Houtmans, 2022). However, original theoretical variations between the measures may represent a methodological confound when investigating associations between self‐compassion and attachment.

Examining self‐compassion via the theoretical lens of attachment requires a concurrent understanding of the interaction between the developmental antecedents of attachment (e.g., anxious) and the current situational triggers (e.g., a threat). The first posits that early experiences of either secure, anxious, or avoidant attachment impact the subsequent development of self‐compassion (Mikulincer & Shaver, 2003; Neff & Beretvas, 2013). The second indicates that being self‐compassionate during a situational trigger (a threat) depends on attachment dimensions (Johnson & O'Brien, 2013). As above, understanding this connection further may promote innovation about the clinical applications of self‐compassion in fostering balanced emotional reactions and health behavioural changes (Roediger et al., 2018).

### The present study

Overall, attachment seems to play a significant role in shaping the extent to which individuals will be self‐compassionate. Research also indicates that different life experiences, age, and sex can influence self‐compassion and/or attachment dimensions (Chopik et al., 2013; Neff & Vonk, 2009; Wren et al., 2012; Yarnell et al., 2015). Therefore, the primary aim of this meta‐analysis was to assess the association magnitude between self‐compassion and secure, anxious, and avoidant attachment in adults. We first hypothesised that self‐compassion would be positively associated with secure attachment and negatively associated with anxious and avoidant attachment. We second hypothesised that variations in the theoretical underpinnings, constructs, and items of the different measures of attachment might affect the magnitude of associations between self‐compassion and attachment. We therefore conducted moderator analyses to identify whether the magnitude of the proposed associations varied as a function of theoretical (scale) and sample‐related (demographics, life experiences) factors.

## METHOD

We registered the protocol for this meta‐analysis on PROSPERO, yet the current study deviated from the pre‐registration. We planned to examine mental health factors as potential moderator variables, but too few studies included a mental health measure to be able to collect this data. We updated PROSPERO following these deviations (https://www.crd.york.ac.uk/prospero/; registration ID: CRD42022346546). We also used the updated PRISMA guidelines (2020; Page et al., 2021) to present the flow of decision making in the selection and inclusion of papers.

### Literature search

A systematic search was completed, guided by an information scientist, using three online databases (SCOPUS, Web of Science and CINAHL) to identify suitable empirical studies from inception until 14th November 2022. Google Scholar was also searched until the studies ceased to be relevant. Search terms included two concepts: (self‐compassion* or self‐kindness) AND (attachment* or relationship development or relationship style* or parent child relationship).

### Inclusion criteria and data extraction

**FIGURE 1:**
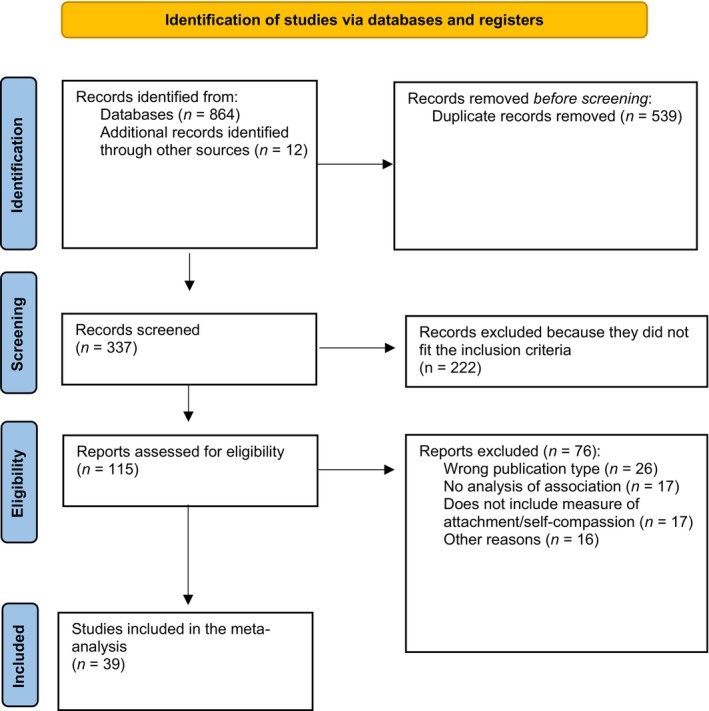
PRISMA flowchart for the selection process of studies meta‐analysed to determine the magnitude of associations between self‐compassion and attachment.

Authors collaboratively established inclusion/exclusion criteria (Table 1). The study selection process is detailed in the PRISMA diagram (Figure 1). In the first phase, CH and VSV independently screened titles, abstracts, and keywords using Rayyan software (Ouzzani et al., 2016), demonstrating excellent inter‐rater reliability (*K* = 0.878, *p* < .001; Cohen, 1992). The second phase involved CH screening full texts of the remaining 115 studies, with VSV independently screening 26% (*k* = 30), achieving moderate agreement (*K* = 0.545, *p* < .001). Three authors (CH, VSV and AT) met to resolve disagreements by referring to the inclusion and exclusion criteria and mutually agreeing whether to include or exclude each paper. Disagreements were linked with misunderstandings of the inclusion criteria from the reviewers. To this end, we decided to further clarify the inclusion and exclusion criteria and reviewed the papers to reach agreement with the presence of a mediator. Authors of studies that included self‐compassion and attachment measures without associative data were contacted by either email or using ResearchGate to request this data if available. Only one author responded but was unable to provide the data in the 2‐week timeframe. CH attempted to retrieve full texts for one study via ResearchGate (D'Alton et al., 2015) but received no response within the response timeframe, leading to their exclusion. The third phase comprised data extraction from 39 included papers, with reference list scanning yielding no additional relevant studies from 12 abstracts reviewed. Three authors were contacted to retrieve data for both attachment dimensions measured by the ECR and ECR‐S, as data was only reported for one (Bistricky et al., 2017; Geller et al., 2021; Raque‐Bogdan et al., 2016). They were given a 2‐week timeframe, but they did not respond.

**TABLE 1:** Study inclusion and exclusion criteria for selection process.

Inclusion criteria	Exclusion criteria
Published journal	Unpublished (e.g., grey publications and dissertations)
Empirical design	Non‐empirical
Inclusion of a validated self‐compassion measure	No inclusion of a validated self‐compassion measure
Inclusion of a validated attachment measure	No inclusion of a validated attachment measure
Analysis of the association between self‐compassion and attachment^a^	No analysis of the association between self‐compassion and attachment^a^
Inclusion of participants either in their late adolescence or adulthood (age 16 or older)	Participants in their childhood or early adolescence
Attachment and self‐compassion measured for the same individual	Attachment and self‐compassion measured for different individuals
English	Non‐English

^a^ We contacted the authors of studies with both attachment and self‐compassion measures to find out if they have correlation data available.

The first author (CH) extracted relevant data from the 39 research studies and recorded them in a coding sheet. Extracted data encompassed (a) publication details (authors, year, and country), (b) methodological details (study design, sample size, sample status, self‐compassion and attachment measures), (c) statistical data (indices and effect sizes for self‐compassion and each attachment dimension), and (d) moderator variables (refer to Table 2). Moderator variables aimed to explore potential sources of variability including average sample age, percentage of females, university student representation (yes/no), and the attachment measure used. Cross‐validation involved the second author (VSV) independently extracting data for 35% of the studies. The coding agreement rate was 89.1%, with discrepancies resolved through discussions with the mediator via a meeting, AT (average *K* = 0.601, see Table 3).

**TABLE 2:** Summary of study characteristics and moderator variables.

Study authors and year	Country	Design, participants, and sampling method	Mean age (*SD*)	Self‐compassion measure	Attachment measure	Attachment dimensions
Amani and Khosroshahi (2020)	Iran	Correlational study *N* = 600 (50% female) Sample of adults in a relationship	35.7	SCS‐SF	AAS (General attachment)	Secure
Arambasic et al. (2019)	Australia	Cross‐sectional study *N* = 92 (100% female) Sample of women with previous diagnosis of breast cancer	58.46 (8.77)	SCS	ECR (General attachment)	Anxious Avoidant
Barnes and Mongrain (2020)	Canada	Experimental study *N* = 4374 (79.6% female) Population‐based sample of adults	33.5 (11.4)	SCS	ECR (General attachment)	Anxious Avoidant
Beard et al. (2017)	UK	Cross‐sectional study *N* = 139 (0% female) Sample of self‐identifying gay male adults	38.3	SCS	ECR‐S (General attachment)	Anxious Avoidant
Bistricky et al. (2017)	USA	Cross‐sectional study *N* = 132 (86.4% female) Sample of adults who have experienced trauma	35.7 (12.2)	SCS‐SF	ECR‐S (General attachment)	Avoidant
Bolt et al. (2019)	UK and USA	Cross‐sectional study *N* = 342 (62.6% female) Population‐based sample of adults in a relationship	27.1 (8.8)	SCS‐SF	ECR‐S (No statement on attachment figure)	Anxious Avoidant
Brophy et al. (2020)	Germany	Cross‐sectional study *N* = 2253 (53.4% female) Population‐based sample of adults	50.32 (17.27)	SCS	AAS (No statement on attachment figure)	Anxious Avoidant
Bugay‐Sökmez et al. (2021)	Turkey	Correlational study *N* = 510 (57.3% female) Sample of university students	21.8 (2.29)	SCS‐SF	ECR‐S (No statement on attachment figure)	Anxious Avoidant
Carbonneau et al. (2021)	Canada	Correlational study *N* = 201 (100% female) Population‐based sample of female adults	25.1 (4.6)	SCS‐SF	ECR‐S (Romantic attachment)	Anxious Avoidant
Cassidy and McLaughlin (2024)	UK	Correlational study *N* = 255 (100% female) Sample of mothers of children with cancer	36.4 (6.8)	SCS	Child–Parent Relationship Scale	Closeness
Davarinejad et al. (2022)	Iran	Correlational study *N* = 304 (100% female) Population‐based sample of divorced females	34.3 (6.5)	SCS‐SF	RAAS (No statement on attachment figure)	Secure Anxious Avoidant
Ding and Xu (2021)	China	Cross‐sectional study *N* = 308 (56% female) Population‐based sample of adults aged 50+	63.02 (7.15)	SCS‐SF	ECR (Romantic attachment)	Anxious Avoidant
Dudley et al. (2018)	UK	Cross‐sectional study *N* = 128 (73% female) Sample of adults who hear voices	37.6	SCS	RQ (No statement on attachment figure)	Secure Preoccupied Dismissing
Fuochi et al. (2018)	Italy	Correlational study *N* = 146 (47.9%) Population‐based sample of adults	33.53 (8.19)	SCS‐SF	ECR‐S (No statement on attachment figure)	Anxious Avoidant
Geller et al. (2021)	Israel	Correlational study *N* = 597 (59.1% female) Population‐based sample of adults	29.45 (9.25)	SCS	ECR (Romantic attachment)	Anxious
Gilbert et al. (2011)	UK	Correlational study *N* = 222 (75.7% female) Sample of university students	22.7 (7.07)	SCS	AAS (No statement on attachment figure)	Closeness Depend Anxious
Holt (2014)	USA	Longitudinal study *N* = 204 (58.0% female) Sample of first‐year undergraduate students	18.1 (0.60)	SCS‐SF	Inventory of Parent and Peer Attachment (Attachment to parents)	Secure
Homan (2018)	USA	Cross‐sectional study *N* = 126 (70.6% female) Population‐based sample of adults aged 60+	70.4 (8.14)	SCS‐SF	ECR (General attachment)	Anxious Avoidant
Huang and Berenbaum (2017)	USA	Cross‐sectional study *N* = 195 (61.3% female) Sample of undergraduate university students	19.3 (1.2)	SCS	ECR‐RS (General attachment)	Anxious Avoidant
Huynh et al. (2022)	USA	Cross‐sectional study *N* = 318 (50% female) Sample of couples in a relationship	29.6 (4.4)	SCS	RSQ	Anxious Avoidant
Kotera and Rhodes (2019)	UK	Cross‐sectional study *N* = 104 (49% female) Population‐based sample of adults	46.3 (12.7)	SCS‐SF	AAS (General attachment)	Close Dependent Anxious
Mackintosh et al. (2018)	UK	Cross‐sectional study *N* = 74 (59.5% female) Sample of clinical population with anxiety and/or depression	40.3 (12.0)	SCS	ECR (Romantic attachment)	Anxious Avoidant
Miyagawa and Kanemasa (2022)	Japan	Cross‐sectional study *N* = 513 (53% female) Population‐based sample of adults in a relationship	26.1 (5.2)	SCCS‐L	ECR‐RS (Romantic attachment)	Anxious Avoidant
Moreira et al. (2015)	Portugal	Cross‐sectional study *N* = 171 (100% female) Population‐based sample of mothers	40.76 (5.36)	SCS	ECR‐RS (Attachment with mother)	Anxious Avoidant
Moreira et al. (2016)	Portugal	Cross‐sectional study *N* = 290 (100% female) Sample of mothers of school‐aged children	41.66 (5.42)	SCS	ECR‐RS (Attachment with mother)	Anxious Avoidant
Murray et al. (2021)	UK	Cross‐sectional study *N* = 148 (72.3% female) Population‐based sample of adults and first‐year psychology students	34.62 (13.46)	SCS	ECR (Romantic attachment)	Anxious Avoidant
Naismith et al. (2019)	UK	Correlational study *N* = 53 (83.0% female) Sample from a clinical population with a personality disorder	32.0 (11.1)	SCS‐SF	ECR‐S (Romantic attachment)	Anxious Avoidant
Neff and McGehee (2010)	USA	Cross‐sectional study *N* = 287 (57% female) Sample of college students	21.1	SCS	RQ (No statement on attachment figure)	Secure Preoccupied Dismissing
Øverup et al. (2017)	USA	Cross‐sectional study *N* = 370 (77.5% female) Sample of university students	22.31 (5.24)	SCS‐SF	ECR (General attachment)	Anxious Avoidant
Pepping et al. (2013)	Australia	Cross‐sectional study *N* = 329 (73.3% female) Sample of undergraduate university students	21.53 (6.59)	SCS	ECR (No statement on attachment figure)	Anxious Avoidant
Quinlan et al. (2022)	Canada	Cross‐sectional study *N* = 52 (41.2% female) Sample of ‘high risk’ young adults	20.86 (2.44)	SCS	ECR (No statement on attachment figure)	Anxious Avoidant
Raque‐Bogdan et al. (2011)	USA	Cross‐sectional study *N* = 208 (73.6% female) Sample of undergraduate university students	20.0 (1.6)	SCS	ECR (Romantic attachment)	Anxious Avoidant
Raque‐Bogdan et al. (2016)	USA	Cross‐sectional study *N* = 1306 (100% female) Sample of first‐year university students	18.73 (2.77)	SCS	ECR‐RS (Attachment with mother selected)	Anxious
Reizer (2019)	Israel	Correlational study *N* = 202 (73.0% female) Sample of service‐sector employees	27.93 (9.12)	SCS	ECR‐S (Attachment with close relationships)	Anxious Avoidant
Set (2021)	Germany	Correlational study *N* = 203 (66.0% female) Sample of university students	20.73 (2.86)	SCS	ECR (General attachment)	Anxious Avoidant
Valikhani et al. (2018)	Iran	Correlational study *N* = 392 (49.2% female) Sample of university students	24.75 (3.74)	SCS‐SF	RAAS (General attachment)	Insecure
Wei et al. (2011)	USA	Cross‐sectional study *n* = 195 (55.0% female) Sample of university students *n* = 136 (43.0% female) Population‐based sample of adults in a relationship	20.07 (2.77) 43.44 (10.22)	SCS	ECR (General attachment)	Anxious Avoidant
Yang et al. (2022)	China	Cross‐sectional study *N* = 301 (41.5% female) Sample of adults at the end‐stage of renal disease	51.98 (14.31)	SCS	ECR‐S (No statement on attachment figure)	Anxious Avoidant
Zhang and Chen (2017)	USA	Experimental study *N* = 153 (49.0% female) Sample of adults from Amazon's Mechanical Turk	32.67 (10.55)	SCS	ECR‐S (No statement on attachment figure)	Anxious Avoidant

Abbreviations: AAS, Adult Attachment Scale; ECR, Experiences in Close Relationships Scale; ECR‐RS, Relationship Structures Questionnaire; ECR‐S, Experiences in Close Relationships Scale‐ Short Form; RAAS, Revised Adult Attachment Scale; RQ, Relationships Questionnaire; RSQ, Relationship Scales Questionnaire;SCS, Self‐Compassion Scale; SCS‐SF, Self‐Compassion Scale‐Short Form; SSCS‐L, State Self‐Compassion Scale‐Long Form.

**TABLE 3:** Inter‐rater agreement for extracted data.

Variable	Percentage agreement (%)	Kappa (*K*)
Study design	86.6	0.595
Sample size *n*	86.6	0.423
Sample status	93.3	0.634
Self‐compassion measure	100.0	1.000
Attachment measure	100.0	1.000
Effect sizes for associations	80.0	0.242
Percentage female	80.0	0.471
Mean age	86.6	0.444
Mean inter‐rater agreement	89.1	0.601

### Quality appraisal

The methodological quality of the 39 studies in the meta‐analysis was assessed using the Appraisal tool for Cross‐Sectional Studies (AXIS: Downes et al., 2016). The AXIS, lacking a quantitative metric, relies on a qualitative assessment of 20 questions (Downes et al., 2016). CH and OH independently completed the AXIS for study evaluation. Inter‐rater agreement was measured using Cohen's kappa coefficient (Cohen, 1992).

### Analyses

Three meta‐analyses investigated the association between self‐compassion and either anxious, avoidant, or secure attachment (Kappen et al., 2018) using Comprehensive Meta‐Analysis (CMA) software (Borenstein et al., 2021). The random effects method was applied for all three meta‐analyses to establish robustness against heterogeneity issues (Field & Gillett, 2010).

All retrieved studies reported the effect sizes as Pearson's *r* value. Each meta‐analysis adhered to Cohen's guidelines to investigate the magnitude of effect sizes (Cohen, 1992), ranging between small (0.10), medium (0.30) and large effects (0.50).

Six studies split the Self Compassion Scale (SCS; Neff, 2003b) into either a positive and negative sub‐scale or into the six individual sub‐domains when investigating any association with attachment (Brophy et al., 2020; Davarinejad et al., 2022; Gilbert et al., 2011; Huynh et al., 2022; Naismith et al., 2019; Yang et al., 2022). All six authors were contacted to provide the data to calculate *r*‐values for the total scale for the SCS; only one responded with the relevant data (Brophy et al., 2020). The *r*‐values for the negative sub‐scales of three studies were reverse coded and averaged with the *r‐*values for the positive sub‐scales when inputted into each meta‐analysis. For two studies (Davarinejad et al., 2022; Huynh et al., 2022), the *r‐*values for the three negative sub‐domains (self‐judgement, over‐identification, and isolation) were reverse coded and averaged with the three positive sub‐domains (self‐kindness, common humanity, and mindfulness) to create an overall *r‐*value. Some studies measured attachment dimensions (dependent and insecure) that are inversely associated with those (avoidant and secure) investigated in the current meta‐analysis (Gilbert et al., 2011; Kotera & Rhodes, 2019; Valikhani et al., 2018), and therefore, they were coded in reverse when meta‐analyzed for avoidant and secure attachment.

Two statistical tests estimated the heterogeneity for each of the three meta‐analyses. The Cochran's Chi‐Squared test (*Q*‐statistic) assessed the degree of variability among the studies (Cochran, 1954) where a statistically significant result suggests heterogeneity. The *I* ^2^ statistic calculated the proportion of variance that was due to a real difference across studies rather than random error, using the widely used cut‐offs of 25%, 50%, and 75% to represent low, moderate, and high heterogeneity respectively (Higgins & Thompson, 2002; Slosar, 2009). Moderator analyses were completed to test the hypotheses of the meta‐analysis even if the *Q*‐statistic was non‐significant.

Mixed‐random effects meta‐regressions were conducted to test whether continuous variables, sex (expressed as the per cent female in the sample) and age, moderated the relationship between attachment and self‐compassion. The analyses were completed if there were at least 10 studies in each meta‐analysis, based on guidance to ensure meaningful findings with sufficient statistical power (Deeks et al., 2019). Sample type (university sample versus other) and attachment measurement were each coded as categorical variables, and mixed‐effects methods were used to assess the potential moderating effects of each variable.

Sub‐group analyses were conducted to test variations of both categorical variables if three or more studies were available in each sub‐group, based on recommendations from Card (2015).

#### Risk of bias

Assessment of publication bias determines the likely presence of bias in meta‐analyses due to not including unpublished or unfound research studies. We assessed publication bias using four methods, in line with Card's (2015) recommendation to take a multi‐pronged approach. First, funnel plots of the standard error and sampling variance were visually inspected. Asymmetry in a funnel shape suggests the possibility of publication bias (Card, 2015). Secondly, we used the “trim and fill” method (Duval & Tweedie, 2000) to assess studies that contributed to the asymmetry of the funnel plot. These studies were trimmed, and later reinstated along with imputed values that fill in the funnel plot to accomplish symmetry. These filled results were then compared to the original estimates, and if discrepant this would suggest publication bias. If comparable, then the original results were considered robust to publication bias. Thirdly, Egger's test assesses the asymmetry of the funnel plots (Egger et al., 1997). A statistically significant result (<.05) indicates the likely exclusion of studies reporting a null hypothesis for the association between self‐compassion and attachment (Card, 2015). Lastly, we used P curve analysis (p‐curve app 4.10) to assess publication bias and estimate the true effects of the associations (Simonsohn et al., 2014).

## RESULTS

### Study characteristics

For anxious attachment, there were a total of 34 studies with 35 effect sizes, with a pooled sample size of 15.661 participants, and for avoidant attachment, there were 33 studies with 34 effect sizes, with a pooled sample size of 13.603 participants. For secure attachment, there were nine studies with a pooled sample size of 2.496 participants (see Table 4).

**TABLE 4:** Meta‐analysed effect sizes between self‐compassion and the three attachment dimensions (*k* = 39).

Study	Anxious attachment		Avoidant attachment		Secure attachment	
	*r*	95% CI	*r*	95% CI	*r*	95% CI
Amani and Khosroshahi (2020)	–	–	–	–	.600	[0.546, 0.649]
Arambasic et al. (2019)	−.620	[−0.732, −0.476]	−.630	[−0.739, −0.488]	–	–
Barnes and Mongrain (2020)	−.370	[−0.395, −0.344]	−.270	[−0.297, −0.242]	–	–
Beard et al. (2017)	−.468	[−0.589, −0.327]	−.441	[−0.566, −0.296]	–	–
Bistricky et al. (2017)	–	–	−.459	[−0.584, −0.313]	–	–
Bolt et al. (2019)	−.210	[−0.309, −0.106]	−.120	[−0.221, −0.016]	–	–
Brophy et al. (2020)	−.439	[−0.472, −0.405]	−.451	[−0.483, −0.417]	–	–
Bugay‐Sökmez et al. (2021)	−.610	[−0.662, −0.552]	−.350	[−0.424, −0.271]	–	–
Carbonneau et al. (2021)	−.430	[−0.536, −0.310]	−.400	[−0.510, −0.277]	–	–
Cassidy and McLaughlin (2024)	–	–	–	–	.520	[0.420, 0.600]
Davarinejad et al. (2022)	−.237	[−0.340, −0.128]	−.278	[−0.379, −0.171]	.018	[−0.095, 0.130]
Ding and Xu (2021)	−.224	[−0.328, −0.115]	−.118	[−0.227, −0.006]	–	–
Dudley et al. (2018)	−.260	[−0.415, −0.091]	.130	[−0.045, 0.297]	.380	[0.221, 0.519]
Fuochi et al. (2018)	−.280	[−0.423, −0.123]	−.290	[−0.432, −0.134]	‐	‐
Geller et al. (2021)	−.340	[−0.409, −0.267]	–	–	–	–
Gilbert et al. (2011)	−.510	[−0.601, −0.406]	−.205	[−0.328, −0.075]	.295	[0.170, 0.411]
Holt (2014)	–	–	–	–	.120	[−0.018, 0.253]
Homan (2018)	−.600	[−0.701, −0.475]	−.490	[−0.612, −0.345]	–	–
Huang and Berenbaum (2017)	−.310	[−0.432, −0.177]	−.160	[−0.294, −0.020]	–	–
Huynh et al. (2022)	−.218	[−0.320, −0.111]	−.273	[−0.372, −0.168]	–	–
Kotera and Rhodes (2019)	−.500	[−0.632, −0.340]	−.590	[−0.698, −0.456]	.630	[0.505, 0.729]
Mackintosh et al. (2018)	−.247	[−0.450, −0.020]	−.255	[−0.457, −0.028]	–	–
Miyagawa et al. (2022)	−.357	[−0.430, −0.279]	−.236	[−0.316, −0.153]	–	–
Moreira et al. (2015)	−.300	[−0.431, −0.157]	−.340	[−0.466, −0.200]	–	–
Moreira et al. (2016)	−.180	[−0.289, −0.066]	−.210	[−0.317, −0.097]	–	–
Murray et al. (2021)	−.450	[−0.570, −0.311]	−.230	[−0.377, −0.071]	–	–
Naismith et al. (2019)	−.065	[−0.329, 0.209]	−.164	[−0.416, 0.111]	–	–
Neff and McGehee (2010)	−.230	[−0.337, −0.117]	.050	[−0.066, 0.165]	.390	[0.287, 0.484]
Øverup et al. (2017)	−.510	[−0.582, −0.430]	−.340	[−0.427, −0.247]	–	–
Pepping et al. (2013)	−.350	[−0.441, −0.251]	−.190	[−0.292, −0.084]	–	–
Quinlan et al. (2022)	−.551	[−0.716, −0.327]	−0.487	[−0.671, −0.247]	–	–
Raque‐Bogdan et al. (2011)	−.434	[−0.538, −0.317]	−.188	[−0.316, −0.053]	–	–
Raque‐Bogdan et al. (2016)	−.380	[−0.425, −0.333]	–	–	–	–
Reizer (2019)	−.420	[−0.527, −0.299]	−.360	[−0.474, −0.234]	–	–
Set (2021)	−.320	[−0.438, −0.191]	−.120	[−0.254, −0.018]		
Valikhani et al. (2018)	–	–	–	–	.470	[0.060, 0.314]
Wei et al. (2011)^a^	−.380	[−0.494, −0.253]	−.360	[−0.498, −0.204]	–	–
Wei et al. (2011)^b^	−.380	[−0.515, −0.226]	−.150	[−0.285, −0.010]	–	–
Yang et al. (2022)	−.440	[−0.527, −0.344]	−.320	[−0.418, −0.215]	–	–
Zhang and Chen (2017)	−0.580	[−0.676, −0.464]	−0.320	[−0.456, −0.170]	–	–
*k*	35		34		9	
Meta‐analyses results						
Average *r*, 95% CI	−.383	[−0.419, −0.346]	−.282	[−0.329, −0.233]	.395	[0.248, 0.524]

^a^ University sample.

^b^ Community sample.

Most studies were conducted in the USA (*k* = 12), followed by the UK (*k* = 9), with the remaining studies carried out in other countries. All studies relied on opportunistic sampling, with 13 recruiting participants from universities, six studies recruiting from clinical/health populations, and the remaining relying on community samples or participants representative of the general population. The range of the average ages of participants in each study varied between 18.1 and 70.4 years. There was a disproportionate representation of females in the meta‐analyses, with 30 studies recruiting either females only or more females than males, equating to 66.0% of the total pooled sample sizes of all studies.

All 39 studies measured self‐compassion using the Self Compassion Scale, with 22 adopting the original version (Neff, 2003b), 14 using the short form (Raes et al., 2011), and one including the ‘state’ version (Neff et al., 2021). Most studies (*k* = 28) measured attachment with the Experience in Close Relationship Scale (ECR; Brennan et al., 1998a), 10 opting for a short version and five opting for the Relationships Structures Questionnaire (Fraley, Heffernan, et al., 2011; Fraley, Vicary, et al., 2011; Wei et al., 2007). Whereas few studies adopted alternative measures, including the Adult Attachment Scale (*k* = 6, AAS; Collins & Read, 1990), the Relationship Questionnaires (*k* = 2, RQ; Bartholomew & Horowitz, 1991), the Relationship Scales Questionnaire (*k* = 1, RSQ; Griffin & Bartholomew, 1994), the Child Parent Relationship Scale (*k* = 1, Driscoll & Pianta, 2011) and the Inventory of Parent and Peer attachment assessment (*k* = 1, IPPA; Armsden & Greenberg, 1987).

### Methodological quality

Twenty‐eight studies showed consistent quality across at least 15 of the 20 questions based on the two authors' individual assessments. The quality assessment of only two studies suggested concern across multiple questions (Gilbert et al., 2011; Neff & McGehee, 2010). Questions related to the justification of the sample size, the management of non‐responders, and the response rate were the main quality issues across most studies in the meta‐analyses. Inter‐rater agreement was moderate, *K* = 0.555.

### Meta‐analyses of self‐compassion and attachment dimensions

Table 4 presents the correlations, confidence intervals (95%), and the results from the meta‐analyses conducted on self‐compassion and each of the three attachment dimensions. The meta‐analysis of self‐compassion and anxious attachment revealed a significant negative average association, with a medium effect size (*r*_avg_ = −.383, 95% CI [−0.419, −0.346], *p* < .001). The *Q* test of heterogeneity of the effect sizes was significant, *Q*_total_ (34) = 193.788, *p* < .001; *I* ^2^ = 82.455%. The meta‐analysis between avoidant attachment and self‐compassion revealed a significant negative average association, with a small effect size (*r*_avg_ = −.282, 95% CI [−0.329, −0.233], *p* < .001). The *Q* test of heterogeneity of the effect sizes was significant, *Q*_total_ (32) = 249.72, *p* < .001; *I* ^2^ = 86.785%. The meta‐analysis between self‐compassion and secure attachment revealed a positive average association, with a medium effect size (*r*_avg_ = .395, 95% CI [0.248, 0.524], *p <* .001). The *Q* test of heterogeneity of the effect sizes was significant, *Q*_total_ (7) = 132.578, *p* < .001; *I* ^2^ = 93.966%. Moderator analyses were therefore conducted for each of the three attachment dimensions to examine potential sources of the high heterogeneity.

### Moderator analysis of self‐compassion and anxious attachment

Meta‐regressions revealed no significant variation in the magnitude of association between self‐compassion and anxious attachment as a function of participant sex, *b* = 0.001 [−0.001, 0.003], *Q*_model_ (1) = 0.350, *p* = .557, or age, *b* = −0.001 [−0.003, 0.003], *Q*_model_ (1) = 0.030, *p* = .871. We conducted a moderator analysis of the effect of university/college students (*k* = 10) versus non‐student samples (*k* = 24), excluding one study (Murray et al., 2021) due to incomplete data on student proportion. Associations between self‐compassion and anxious attachment did not differ for studies that used university/college student samples, *r*_avg_ = −.412, versus non‐student samples, *r*_avg_ = −.366, *Q*_value_ (1) = 1.051, *p* = .305. Moderator analysis showed associations between self‐compassion and anxious attachment did not significantly vary based on the attachment measure used, *Q*_value_ (1) = 0.730, *p* = .393, with studies that used the ECR having comparable effect sizes (*r*_avg_ = −.393; *k* = 28) to studies that used alternative measures of attachment (*r*_avg_ = −.347; *k* = 7).

### Moderator analysis of self‐compassion and avoidant attachment

As expected, meta‐regression revealed significant variation in associations between self‐compassion and avoidant attachment as a function of age, *b* = −0.004 [−0.008, −0.001], *Q*_model_ (1) = 6.230, *p* = .012. The results suggest that as the mean age of the samples increased, the negative association between the two constructs also increased. In contrast, there was no significant variation in the associations between the two constructs as a function of participants' sex, *b* = 0.001 [−0.001, 0.003], *Q*_model_ (1) = 0.001, *p* = .964.

Sub‐group analysis of the sample type revealed significant variation in the association between self‐compassion and avoidant attachment, *Q*_value_ (1) = 6.205, *p* = .013. Studies with non‐student samples (*k* = 24; *r*_avg_ = −.321) showed larger effect sizes than studies with university/college student samples (*k* = 9; *r*_avg_ = −.189), excluding one study (Murray et al., 2021) due to incomplete data on student proportion. Subgroup analysis revealed the association between self‐compassion and avoidant attachment did not vary significantly as a function of attachment measure, *Q*_value_ (1) = 0.186, *p* = .666, with studies (*k* = 27) that used the ECR (or adapted versions; *r*_avg_ = −.286) having comparable effects to those that used alternative measures of attachment (*k* = 7, *r*_avg_ = −.246).

### Moderator analysis of self‐compassion and secure attachment

Less than 10 studies (*k* = 9) examined associations between self‐compassion and secure attachment, indicating that meta‐regressions to test the potential influences of age and sex were not viable. Further ad‐hoc exploratory sub‐group analyses, assessing the influence of sample type, revealed no significant variation in effect sizes, *Q* value (1) = 0.740, *p* = .390, with studies that used university/college student samples (*r*_avg_ = .329, k = 4) having comparable effects to those that used non‐student samples (*r*_avg_ = .449, *k* = 5). There were too few studies for a sub‐group analysis to assess the role of measures of attachment, comparing the AAS (*k* = 5), RQ (*k* = 2), IPPA (*k* = 1) and Child Parent Relationship Scale (*k* = 1).

### Sensitivity analysis

Sensitivity analyses were conducted to ensure the results were robust to methodological variances. Each meta‐analysis was re‐run with every study removed individually to test if any single study defined the summary effect sizes. The summary effect size remained consistent after the removals of every study for each of the three targeted variables: anxious attachment (*r*_avg_ between −.390 and −.370), avoidant attachment (*r*_avg_ between −.291 and −.270), and secure attachment (*r*_avg_ between .360 and .440). Although most studies measured general attachment in adulthood, we ran additional analyses excluding studies that measured attachment to a parent figure (Holt, 2014; Moreira et al., 2015, 2016; Raque‐Bogdan et al., 2016). The overall meta‐analyses remained essentially unchanged after removing three studies for anxious attachment, *r*_avg_ = −.393 [−0.431, −0.353], after removing two studies for avoidant attachment, *r*_avg_ = −.283 [−0.332, −0.232], and after removing one study for secure attachment, *r*_avg_ = .426 [0.280, 0.553].

### Publication bias

**FIGURE 2:**
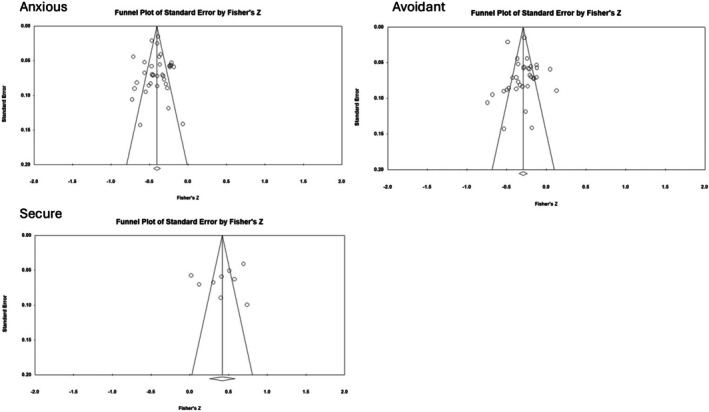
Funnel plots for effect sizes for standard error for each attachment dimension.

The asymmetry in the funnel plots observed for all three meta‐analyses appeared marginal, see Figure 2. The likelihood of publication bias was low, as supported by the trim and fill analysis. A total of one, three, and two studies were trimmed, and similar summary effect sizes were created when imputed effects were added to the anxious attachment, *r*_avg_ = −.383 [−0.419, −0.346], avoidant attachment, *r*_avg_ = −.311 [−0.360, −0.260], and secure attachment meta‐analyses, *r*_avg_ = .483 [0.324, 0.616], respectively. The Egger's tests of the intercept were non‐significant for anxious attachment, *b*_0_ = 0.072 [−1.457, 1.602], *t* (33) = 0.096, *p* = .924, avoidant attachment, *b*_0_ = 0.634 [−1.126, 2.394], *t* (32) = 0.733, *p* = .469, and secure attachment, *b*_0_ = −4.654 [−17.890, 8582], *t* (7) = 0.875, *p* = .433, also suggesting the absence of publication bias. P curve analyses reported right‐skewed p curves suggesting a true effect for each association (anxious, avoidant and secure) with self‐compassion (Figures S1–S3).

## DISCUSSION

Examining the association between self‐compassion and different attachment dimensions may shed light on why some individuals exhibit self‐compassion when confronting challenging events (Germer, 2009), whilst others struggle to self‐soothe in similar events (Neff, 2003a). The current study contributes both theoretically and clinically to the current understanding of self‐compassion and attachment by demonstrating that self‐compassion, a trainable skill, is linked to effective responses to threats. The meta‐analyses found that secure attachment was associated with high levels of self‐compassion, indicating a likely capacity to practice self‐compassion in the face of threats. Anxious and avoidant attachment dimensions were associated with low levels of self‐compassion, suggesting limits in capacity for self‐compassion when in stressful environments. The links of self‐compassion with secure and anxious attachment were robust to the moderators tested. However, the associations with avoidant attachment varied by age and sample type (community vs. university/college samples), indicating a larger magnitude of negative associations in older adults and community samples.

These findings are consistent with a recent scoping review that found negative associations for self‐compassion with anxious and avoidant attachment and a positive association with secure attachment (Lathren et al., 2021). They are also consistent with another meta‐analysis, linking self‐compassion with childhood neglect (Zhang et al., 2022), a key indicator of avoidant attachment (Grady & Shields, 2018).

The findings also align with the relevant theory explaining how attachment dimensions can impact the use of self‐compassion (Neff, 2003a; Phillips & Hine, 2021). Secure attachment fosters effective distress management and confidence in handling unpredictable events (Mikulincer, 1998; Mikulincer et al., 1993). Individuals with secure attachment, having received support from caregivers, can adeptly practice self‐kindness in moments of distress (Mikulincer & Shaver, 2004). Such individuals may also demonstrate awareness of shared human experiences during distress, acknowledging imperfections as common limitations, rather than feeling isolated in their suffering. These responses correspond to the three facets of self‐compassion—mindfulness, self‐kindness, and recognising common humanity (Neff, 2003a) but may be reduced with insecure attachment.

In anxious attachment, individuals struggle to be mindful due to emotional regulation difficulties (Mikulincer, 1998; Pepping et al., 2013, 2015; Schore, 2017), often leaning towards self‐criticism (Cantazaro & Wei, 2010) and feelings of shame (Muris et al., 2014). When recognising their own failures, they tend to criticise themselves and rely on the protection, approval, and care from others rather than recognising failures and imperfections as common human experiences (Caldwell & Shaver, 2015; Joeng et al., 2017; Sagone et al., 2023). Similarly, individuals who rely on avoidant attachment present maladaptive emotional regulation patterns, encompassing either overt self‐reliance (Mikulincer et al., 2009) or deactivation (e.g., people avoiding noticing their own emotions; Shaver & Mikulincer, 2007).

With regards to the moderator variables, previous research reports variations in both attachment dimensions and self‐compassion, depending on either sex or age (Chopik et al., 2013; Del Giudice, 2009; Hwang et al., 2016; Neff & Vonk, 2009; Scharfe, 2016; Souza & Hutz, 2016; Wren et al., 2012). Associations between self‐compassion and attachment did not vary based on the proportion of females assessed in the current meta‐analysis, suggesting potential generalisation across sexes for this association. Similarly, although previous research indicates that age affects the two constructs separately (Neff & Vonk, 2009; Wren et al., 2012), we found no variation in the magnitude of associations between self‐compassion and anxious attachment, based on age. It may be that the association remains steady across the lifespan, but longitudinal analyses are needed to support this. In contrast, we found that the negative association between self‐compassion and avoidant attachment increased with age. It is possible that life events and milestones (e.g., reduced social support, isolation, and loneliness; Spence et al., 2020) associated with ageing worsen some individuals' ability to practice self‐compassion (e.g., harder to think about and relate to common humanity) with avoidant attachment, a proposition that could be fruitful to explore in future research. Compassion‐focused activities may support the mental health of ageing populations with avoidant attachment styles by enhancing or preserving self‐compassion (Spence et al., 2020).

There was some evidence that the relationship between self‐compassion and attachment varied by sample type, although this was only for avoidant attachment. Effects were lower in university samples compared to non‐student samples, cautioning against generalising research findings from student populations. This discrepancy may reflect age‐related reductions in self‐compassion for those with avoidant attachment, given that university students generally represent a younger demographic. However, unique life experiences either associated with this age trajectory (young adults; Larose & Boivin, 1998) or linked with cohort effects may alter this association as well, highlighting the need to examine how life events may trigger possible changes in emotional regulation responses and attachment dimensions across the lifespan (Bowlby, 1988; Michael Bradley & Cafferty, 2001). No evidence suggested variation in the relationship between self‐compassion and attachment based on the attachment measure used, suggesting generalisability across different measures in research (Brennan et al., 1998b).

### Clinical implications

The findings from this meta‐analysis align with the theoretical suggestions that different dimensions of attachment in early childhood may be the foundation for the formation of adaptive/maladaptive emotional regulatory systems, and thus influence the ability to practice self‐compassion when responding to negative experiences (Gilbert, 2014). Given the relative stability of dimensions of attachment, interventions should focus on helping clients recognise how attachment dimensions drive emotional regulation responses and impact a person's capacity to practice self‐compassion in moments of distress. This can be part of contextual schema therapies (Roediger et al., 2018) that often target these underlying mechanisms.

A bidirectional link between these constructs has recently gained traction in research, which found that self‐compassion may affect attachment dimensions, even early parent–child bonds (Xie et al., 2022). Recent evidence also supports attachment‐based compassion therapy as effective in improving self‐compassion and reducing psychological distress (García‐Campayo et al., 2016; Navarro‐Gil et al., 2020). Future research that takes a longitudinal approach is needed to provide a more nuanced understanding of the bidirectional link between self‐compassion and attachment.

From a public health perspective, the current meta‐analysis supports the need to prevent the development of insecure attachment in early years and the strengthening of avoidant attachment in later life, which may help to promote and preserve self‐compassion and positive health outcomes across the lifespan (Izett et al., 2021; Shirvanian & Michael, 2017; Spence et al., 2020). Indeed, ongoing global efforts persist to improve the early years of life with important human rights and financial implications (Bellis et al., 2023). Our findings further add to the body of evidence supporting continued investment and commissioning in public policy (NICE, 2015; Powell et al., 2021).

### Limitations

The current findings should be considered in light of several limitations. Firstly, certain moderator analyses were hindered by a scarcity of studies (Deeks et al., 2019), preventing an assessment of the relationship between self‐compassion and secure attachment across age, sex, and attachment measures used (Tanaka et al., 2011). Also, only the ECR/ECR‐S could be compared with all other measures of attachment collated together, limiting detailed insight into the potential influence of theoretical variations (e.g., different constructs and items) on the results.

Finally, the present meta‐analysis did not directly compare the different attachment dimensions to indicate differential associations between self‐compassion and attachment dimensions. To investigate the strength and direction of associations in more detail, future studies could adopt a multi‐level mixed effect meta‐analytic design to run further analysis (e.g., with attachment dimension as the moderator). Indeed, further research is necessary to guide clinical practice and determine whether specific attachment dimensions could be useful targets for psychotherapeutic interventions.

## CONCLUSIONS

The findings from the current meta‐analyses show the magnitude of the links between self‐compassion and attachment, indicating a medium positive association with secure attachment, a medium negative association with anxious attachment, and a small negative association with avoidant attachment. Age served as a moderator variable for the link between self‐compassion and avoidant attachment, revealing challenges for older individuals with avoidant attachment in responding to challenges with self‐compassion. The results emphasise the need for further research moving from a focus on self‐compassion dispositional tendencies to self‐compassion interventions for those with insecure attachment. Such research would contribute to our theoretical understanding of the bidirectional and dynamic relationship between the two constructs.

## AUTHOR CONTRIBUTIONS

**Charlotte Hill:** Conceptualization; methodology; software; formal analysis; data curation; investigation; validation; project administration; writing – original draft; writing – review and editing. **Vasilis S. Vasiliou:** Conceptualization; methodology; data curation; investigation; formal analysis; supervision; writing – review and editing; validation. **Fuschia M. Sirois:** Conceptualization; supervision; writing – review and editing; methodology. **Olivia Hughes:** Methodology; writing – review and editing; investigation; validation; formal analysis. **Andrew R. Thompson:** Conceptualization; validation; investigation; supervision; writing – review and editing.

## FUNDING INFORMATION

This manuscript was prepared as part of the partial completion of the first co‐author's doctoral thesis in Clinical Psychology program (D.Clin.Psy) which was conducted at Cardiff University and funded by the NHS Wales, United Kingdom. Equally, this work was completed as part of the second co‐author Post‐doctoral Research Associate Position in Clinical Health Psychology which was funded by the Health Education and Improvement Wales (HEIW).

## CONFLICT OF INTEREST STATEMENT

The authors declare no conflicts of interest.

## Supporting information


Figures S1–S3

## Data Availability

The data that support the findings of this study are available from the corresponding author upon reasonable request.
