# Supplementary material for: A meta‐analysis of self‐compassion and attachment in adults
Source: Psychol Psychother. 2025 Apr 4;98(4):833–58. doi: 10.1111/papt.12590 (PMC12617489; doi:10.1111/papt.12590)
Supplement: Supplementary file 1 — Figures S1–S3 [file PAPT-98-833-s001.docx]

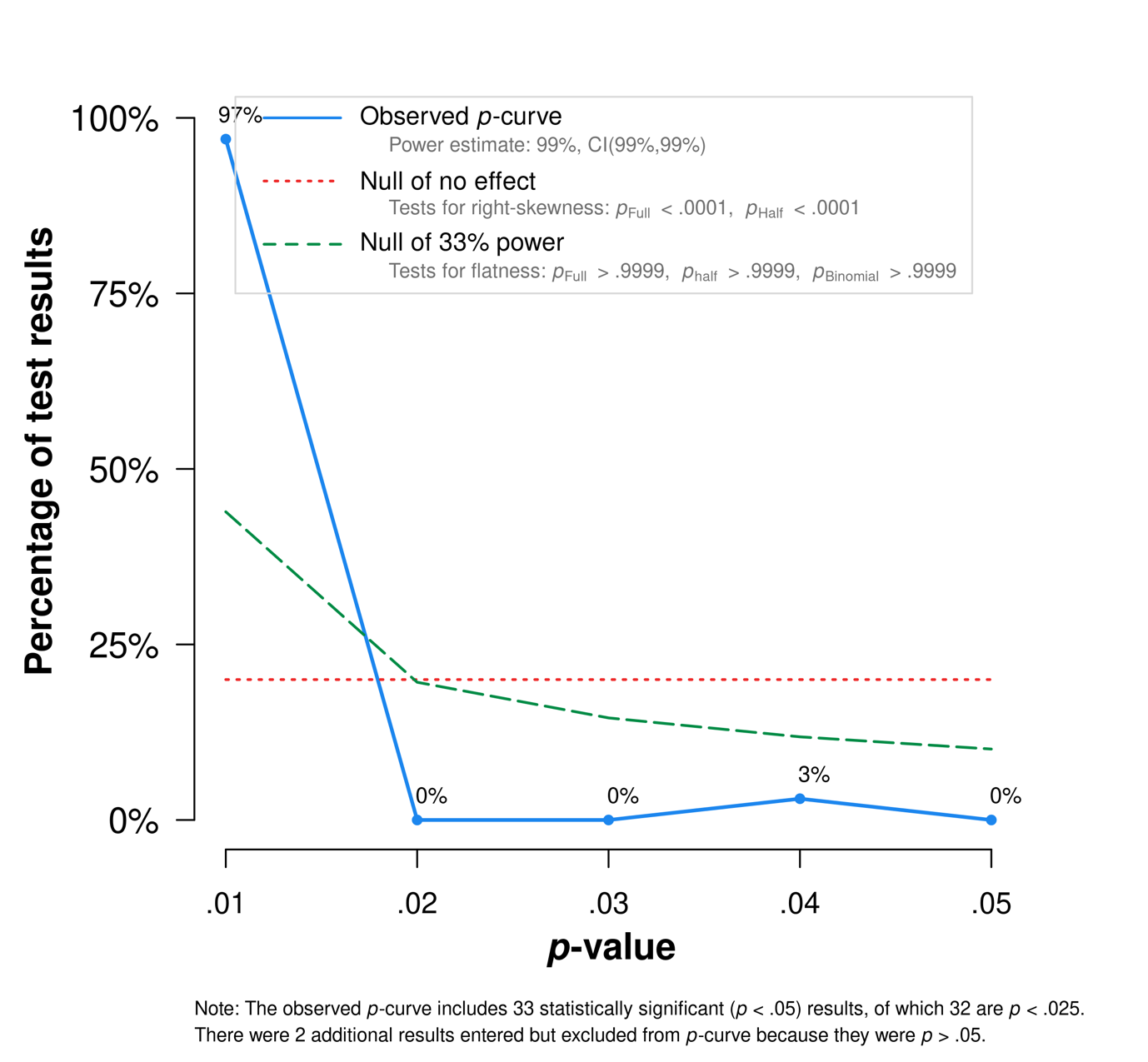


**Figure 1**

*P curve for anxious attachment x self-compassion.*

*X-axis represents P-value and Y-axis represents percentage of the test results. Blue line indicates observed p-value in this meta-analysis. P curve analysis to assess publication bias and true effect of association reported a right-skewed p curve suggesting a true effect of the association.*


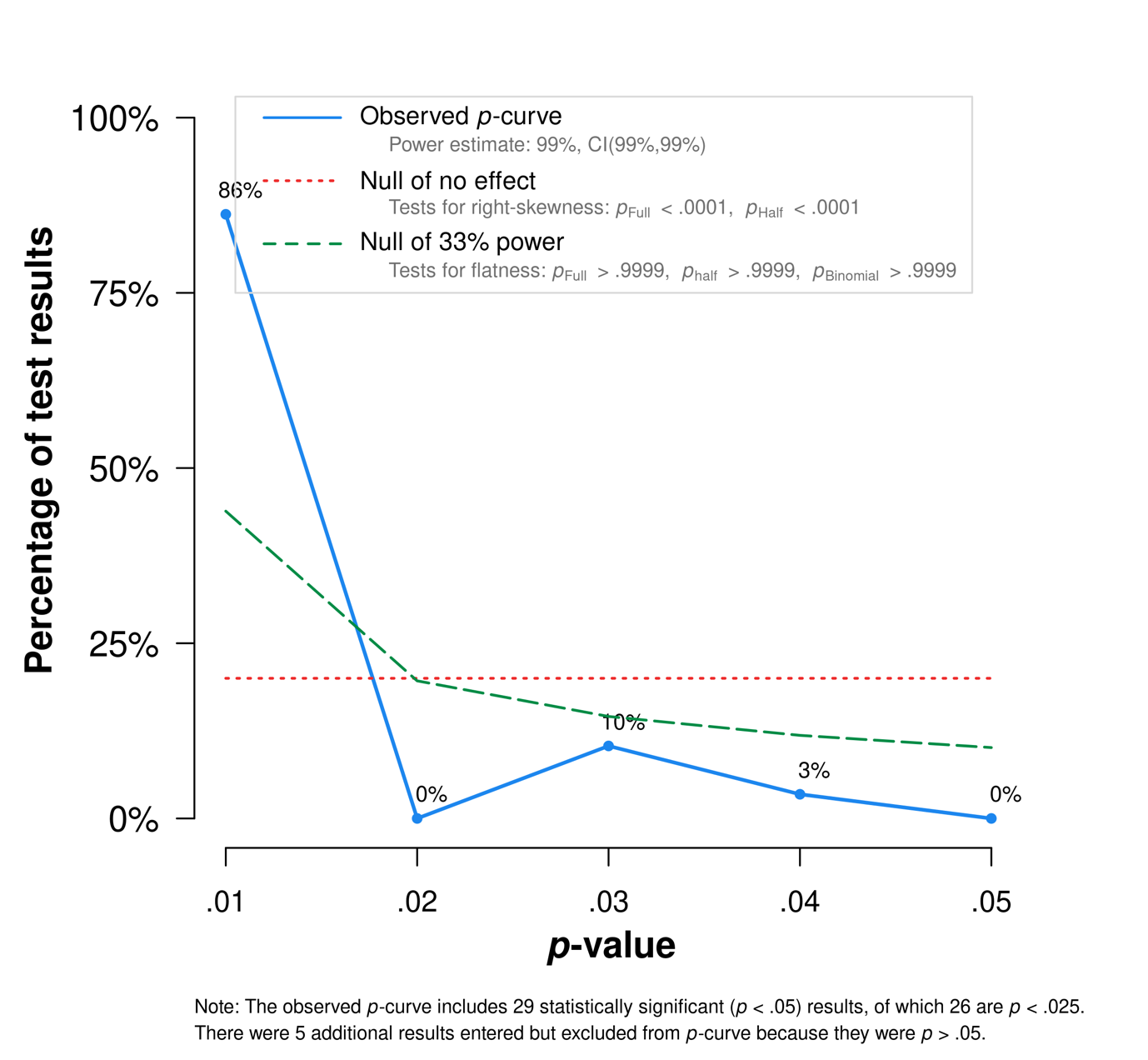


**Figure 2**

*P curve for avoidant attachment x self-compassion.*

*X-axis represents P-value and Y-axis represents percentage of the test results. Blue line indicates observed p-value in this meta-analysis. P curve analysis to assess publication bias and true effect of association reported a right-skewed p curve suggesting a true effect of the association.*


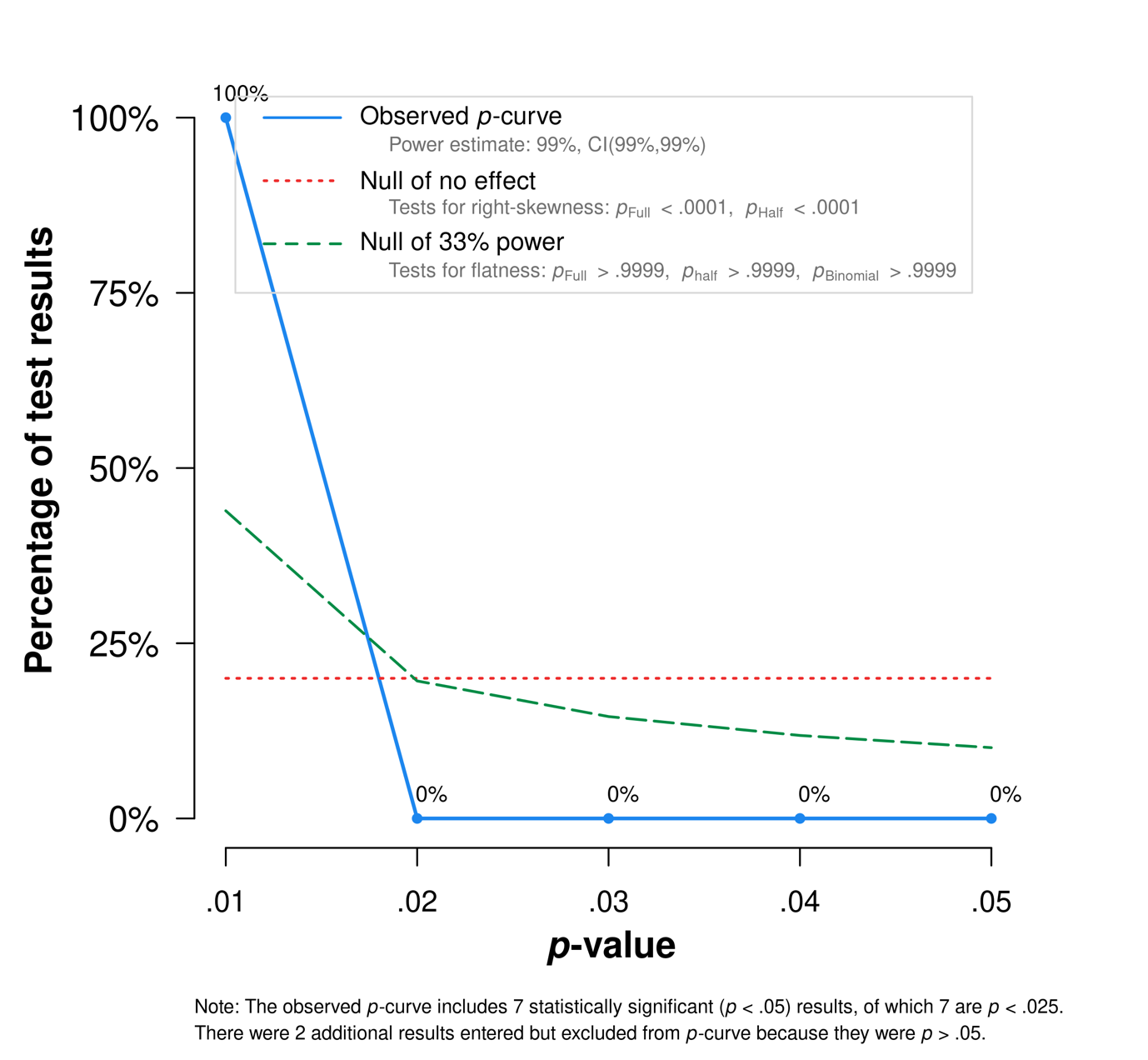
**Figure 3**

*P curve for secure attachment x self-compassion.*

*X-axis represents P-value and Y-axis represents percentage of the test results. Blue line indicates observed p-value in this meta-analysis. P curve analysis to assess publication bias and true effect of association reported a right-skewed p curve suggesting a true effect of the association.*
